# Dipeptidyl peptidase 3 modulates the renin–angiotensin system in mice

**DOI:** 10.1074/jbc.RA120.014183

**Published:** 2020-06-16

**Authors:** Shalinee Jha, Ulrike Taschler, Oliver Domenig, Marko Poglitsch, Benjamin Bourgeois, Marion Pollheimer, Lisa M. Pusch, Grazia Malovan, Saša Frank, Tobias Madl, Karl Gruber, Robert Zimmermann, Peter Macheroux

**Affiliations:** 1 Institute of Biochemistry, Graz University of Technology, NAWI Graz, Graz, Austria; 2 Institute of Molecular Biosciences, University of Graz, NAWI Graz, Graz, Austria; 3 Attoquant Diagnostics GmbH, Vienna, Austria; 4 Gottfried Schatz Research Center, Division of Molecular Biology and Biochemistry, Medical University of Graz, Graz, Austria; 5 Diagnostic & Research Institute of Pathology, Medical University of Graz, Graz, Austria; 6 BioTechMed Graz, Graz, Austria

**Keywords:** angiotensin II, dipeptidyl peptidase 3 (DPP3), metalloprotease, mouse, oxidative stress, peptidase, renal physiology, renin angiotensin system, kidney function, sex-specific difference, reactive oxygen species (ROS)

## Abstract

Dipeptidyl peptidase 3 (DPP3) is a zinc-dependent hydrolase involved in degrading oligopeptides with 4–12 amino acid residues. It has been associated with several pathophysiological processes, including blood pressure regulation, pain signaling, and cancer cell defense against oxidative stress. However, the physiological substrates and the cellular pathways that are potentially targeted by DPP3 to mediate these effects remain unknown. Here, we show that global DPP3 deficiency in mice (DPP3^−/−^) affects the renin–angiotensin system (RAS). LC–MS–based profiling of circulating angiotensin peptides revealed elevated levels of angiotensin II, III, IV, and 1–5 in DPP3^−/−^ mice, whereas blood pressure, renin activity, and aldosterone levels remained unchanged. Activity assays using the purified enzyme confirmed that angiotensin peptides are substrates for DPP3. Aberrant angiotensin signaling was associated with substantially higher water intake and increased renal reactive oxygen species formation in the kidneys of DPP3^−/−^ mice. The metabolic changes and altered angiotensin levels observed in male DPP3^−/−^ mice were either absent or attenuated in female DPP3^−/−^ mice, indicating sex-specific differences. Taken together, our observations suggest that DPP3 regulates the RAS pathway and water homeostasis by degrading circulating angiotensin peptides.

Dipeptidyl peptidase 3 (DPP3, EC 3.4.14.4) is a metalloprotease that specifically cleaves dipeptides at the N-terminus of peptides with 4–12 amino acids. It is ubiquitously expressed in both prokaryotes and eukaryotes. DPP3 is part of the central human proteome, *i.e.* it belongs to a set of proteins ubiquitously and abundantly expressed in all human cells (1). The crystal structures of bacterial, yeast, and human DPP3 have been reported (2–4). All these structures are composed of an upper and a lower domain separated by a wide cleft which has been shown to be the substrate binding site (4, 5). The conserved (HE*XX*GH) and (EECRAE/D) motifs are part of the upper domain and are involved in the coordination of a catalytically essential zinc ion in the binding site (3).

A variety of small bioactive peptides, such as met-enkephalin and angiotensin (I and II), are substrates of DPP3, although the full range of substrate peptides remains undefined (1, 5). Consequently, DPP3 has been implicated in pain modulation (6, 7) and blood pressure regulation (8, 9). In addition, DPP3 exhibits a moonlighting activity in the Keap1 (Kelch-like ECH-associated protein 1)–Nrf2 (nuclear factor erythroid 2-related factor 2) signaling pathway that appears to play a role in stress responses through transcriptional regulation of the antioxidant response element (ARE) (10). Despite the structural and biochemical evidence indicating an intriguing involvement of DPP3 in peptide processing and signaling as well as in the response to oxygen stress, its physiological role and potential involvement in disease-related processes is currently unknown.

Recently, it was reported that adult *DPP3* knockout mice exhibited a growth defect, increased bone loss, and significantly elevated bone marrow cellularity. Deletion of *DPP3* also resulted in oxidative stress and alterations of bone microenvironment favoring osteoclast over osteoblast lineage. The osteoclasts showed increased reactive oxygen species (ROS) production, which made them prone to apoptosis (11). A previous study further established that DPP3 administration to angiotensin II (Ang II)–induced hypertensive mice could significantly diminish systolic blood pressure, cardiac hypertrophy, and myocardial fibrosis to an extent at par with the effect of the angiotensin receptor blocker candesartan. It was also observed that DPP3 effectively reduced urine albumin excretion, kidney damage, and the renal protein levels of the proinflammatory molecule monocyte chemo-attractant protein-1 and the procoagulant platelet activator inhibitor (9). Taken together, DPP3's ability to degrade various bioactive peptides may have complex effects and influence basic physiological processes, particularly those affecting cellular metabolism and oxidative stress.

Ang II, which is the most prominent substrate reported for DPP3, is the principal effector of the renin–angiotensin system (RAS). RAS plays a pivotal role in the pathophysiological modulation of renal and cardiovascular processes (12, 13). Ang II regulates vasoconstriction and is responsible for maintaining homeostasis in the heart and kidney (14). In addition, Ang II is a potent stimulator of NAD(P)H oxidase, which augments formation of ROS in various tissues. Ang II–mediated ROS production has been associated with cell growth, apoptosis, cell migration, and expression of inflammatory and extracellular matrix genes (15). An imbalance between the production of ROS and the antioxidant defense to eliminate these toxic intermediates leads to oxidative stress. There is a plethora of evidence demonstrating the importance of oxidative stress in Ang II–mediated metabolic disorders like hypertension, diabetes mellitus, and chronic kidney disease (16–19). Although blockade of the RAS is the most commonly adopted strategy to slow progression of cardiovascular and associated renal diseases, a better understanding of the novel aspects of the RAS is of paramount importance for the development of innovative therapies that target pathologies inflicted by anomalies of this pathway.

In the present study, we attempted to elucidate the physiological role of DPP3 in the RAS system through characterization of DPP3 knockout mice (DPP3^−/−^). Our observations suggest that DPP3 regulates the RAS pathway and water homeostasis by degrading circulating angiotensin peptides. Interestingly, the lack of DPP3 affected only the phenotype of male mice, with the effects either being absent or much weaker in female mice. This sex-specific difference points at a link between the endocrine system and the physiological role of DPP3. The characterization of DPP3 in this study establishes that it has strong metabolic implications through the modulation of the RAS pathway, a property that could be useful in the management of several cardiovascular and related metabolic pathologies.

## Results

### Generation and gross characterization of DPP3 knockout mice

**Figure 1.**
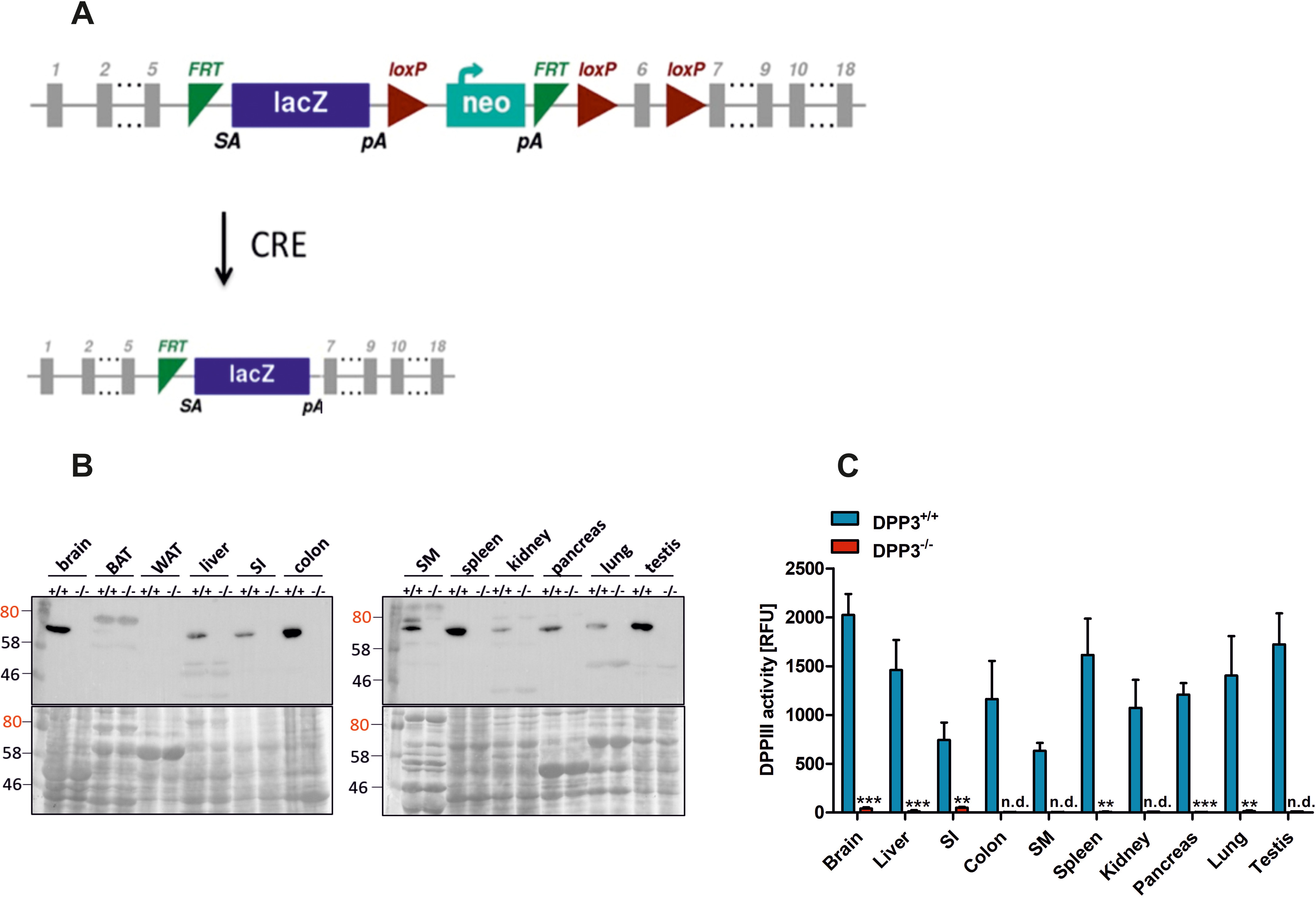
**Generation and validation of DPP3 knockout mice.** *A*, strategy for the generation of *DPP3*-knockout mice. *B* and *C*, demonstration of the lack of DPP3 in male *DPP3*^−/−^ mice by Western blotting (*B*) and activity assays using Arg-Arg-2-naphthylamide as an artificial substrate (*C*). *BAT*, brown adipose tissue; *SI*, small intestine; *SM*, skeletal muscle; *WAT*, white adipose tissue (12–16 weeks of age; *n* = 3/group); *n.d.*, non detectable. **, *p* < 0.01; ***, *p* < 0.001 *versus* WT mice based on unpaired two-sided Student's *t* test. The data are representative of three technical replicates from three biological replicates and presented as means ± S.D.

To investigate the function of DPP3 *in vivo*, we generated mice globally lacking DPP3. The mice were generated using ES cells from EUCOMM containing a β-galactosidase cassette (lacZ) and a promotor-driven selection cassette (neo) between exons 5 and 6 of the *DPP3* gene. The selection cassettes, as well as exon 6 of *DPP3*, were flanked by loxP sites. Mice bearing the targeted allele were crossed with transgenic mice expressing Cre-recombinase under the control of a cytomegalovirus (CMV) promotor, resulting in deletion of neo and exon 6 (Fig. 1*A*). DPP3 was detected by Western blotting in most investigated tissues of WT controls (DPP3^+/+^), but not in tissue lysates of DPP3^−/−^ mice (Fig. 1*B*). A comparison of DPP3 activity in various tissues of DPP3^+/+^ and DPP3^−/−^ mice using the artificial substrate Arg-Arg-2-naphthylamide clearly demonstrated blunted activity in DPP3^−/−^ tissue lysates (Fig. 1*C*).

**Figure 2.**
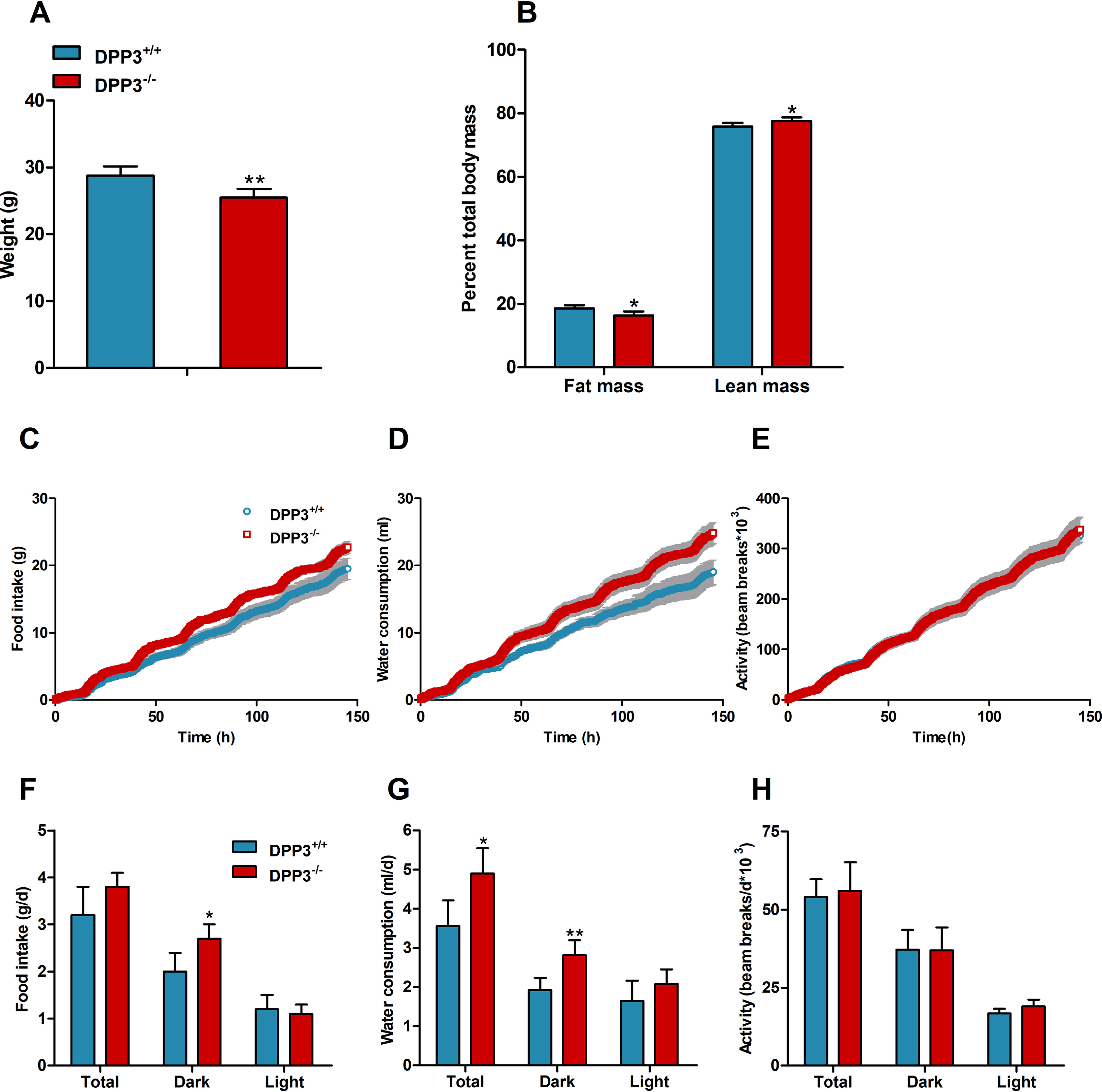
**DPP3^**−/−**^ mice display lower body weight and altered food and water intake.** *A* and *B*, body weight (*A*) and body composition (*B*) of male mice fed a regular chow diet. *C–H*, cumulative (*top panels*) and total (*bottom panels*) food intake (*C* and *F*, respectively), water consumption (*D* and *G*, respectively), and locomotive motion (*E* and *H*, respectively) were measured in metabolic cages over the light and dark phases in male DPP3^−/−^ and DPP3^+/+^ mice (12–16 weeks of age; *n* = 6/group) fed a regular chow diet over a period of 6 consecutive days. *, *p* < 0.05; **, *p* < 0.01 *versus* WT mice based on unpaired two-sided Student's *t* test. The data are representative for two independent cohorts and presented as the means ± S.D.

**Figure 3.**
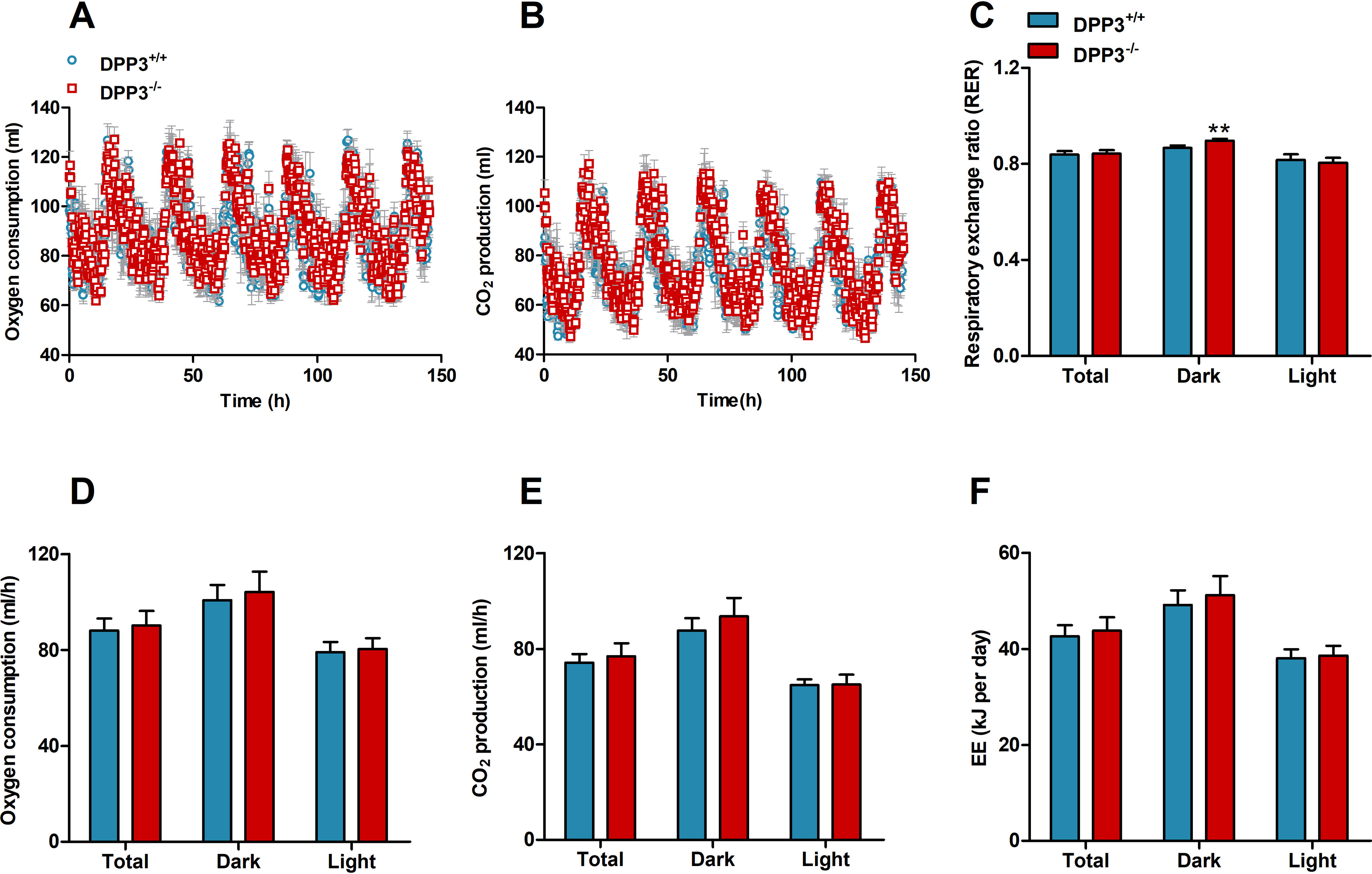
**DPP3 knockout mice display unaltered energy expenditure.** Cumulative and daily oxygen consumption (*A* and *D*, respectively), carbon-dioxide production (*B* and *E*, respectively), respiratory exchange ratio (*RER*, *C*), and EE (*F*) were measured in metabolic cages over the light and dark phases in male DPP3^−/−^ and DPP3^+/+^ mice (12–16 weeks of age; *n* = 6/group) fed a regular chow diet over a period of 6 consecutive days. *, *p* < 0.05; **, *p* < 0.01 *versus* WT mice based on unpaired two-sided Student's *t* test. The data are representative of two independent cohorts and presented as the means ± S.D.

In accordance with a previously published study (11), gross characterization revealed that male DPP3^−/−^ mice exhibit lower body weight (Fig. 2*A*) and less fat mass than WT littermates (Fig. 2*B*). Food and water consumption, as well as energy expenditure and spontaneous locomotor activity, were monitored in metabolic cages over a period of 150 h. Cumulative analysis revealed a slight increase in food intake in male DPP3^−/−^ mice (Fig. 2*C*) caused by increased food consumption during the dark phase (Fig. 2*F*). Cumulative water intake was significantly elevated (Fig. 2*D*) because of a 27 and 46% increase in drinking during the light and dark phase, respectively (Fig. 2*G*). Cumulative (Fig. 2*E*) and daily (Fig. 2*H*) locomotor activity remained unchanged. As shown in Fig. 3 (*A–F*), oxygen consumption and carbon dioxide production were comparable between genotypes, whereas the respiratory exchange ratio was slightly increased in the knockout mice during the dark phase. We also calculated energy expenditure (EE) based on the amount of oxygen consumed and carbon dioxide produced, using the formula: EE (kJ per day) = 15.818*VO_2_ + 5.176*VCO_2_/1000*24 (20) and found that it remained unaltered between the genotypes (DPP3^+/+^, 42.6 ± 2.3 kJ per day *versus* DPP3^−/−^, 43.7 ± 2.8 kJ per day; total energy expenditure).

**Figure 4.**
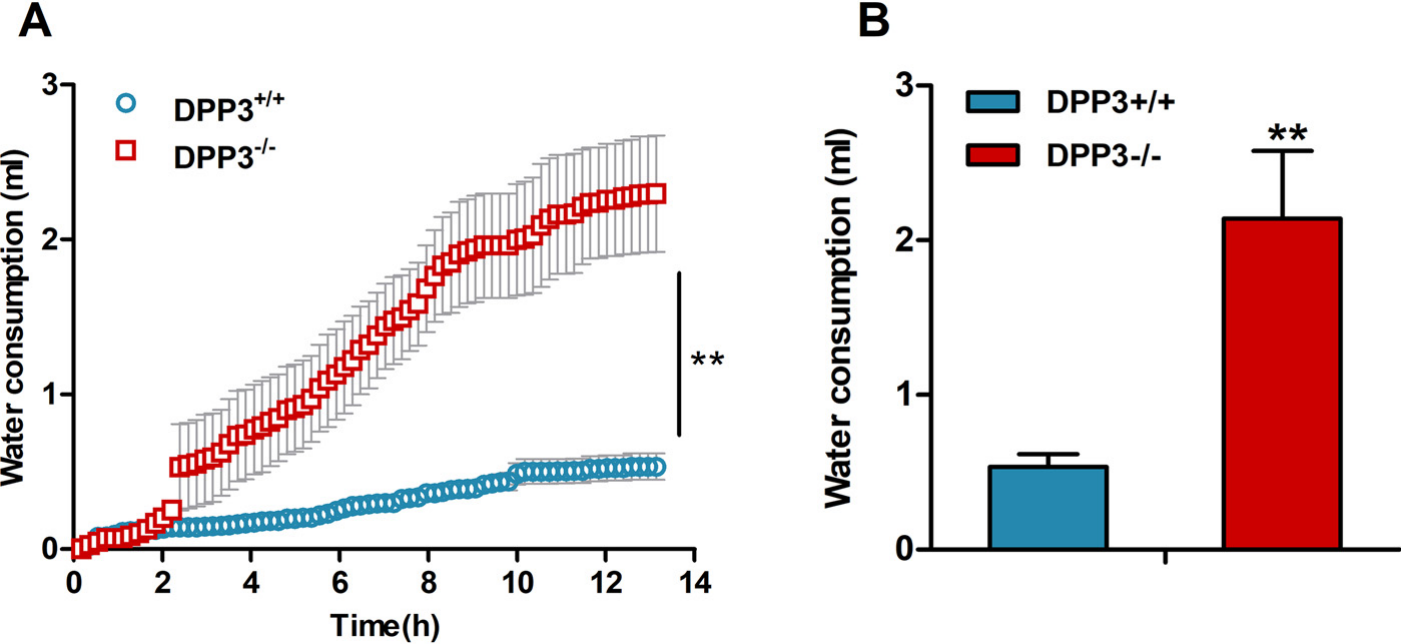
**DPP3^**−/−**^ mice exhibit significantly elevated water consumption during fasting.** *A* and *B*, cumulative (*A*) and daily (*B*) water intake during a 13-h fasting period was measured in metabolic cages in male DPP3^−/−^ and DPP3^+/+^ mice (12–16 weeks of age; *n* = 6/group). *, *p* < 0.05; **, *p* < 0.01 *versus* WT mice based on unpaired two-sided Student's *t* test. The data represent the means ± S.D.

Mice consume substantially lower amounts of water after food deprivation. To investigate whether the drinking behavior of male DPP3^−/−^ mice was also altered upon food restriction, we monitored water consumption during a 13-h fasting period. Under these conditions, water intake of DPP3^−/−^ mice was ∼4-fold higher as compared to DPP3^+/+^ mice, suggesting that DPP3 deficiency is associated with polydipsia in male animals (Fig. 4).

### DPP3 acts as a modulator of the RAS

**Figure 5.**
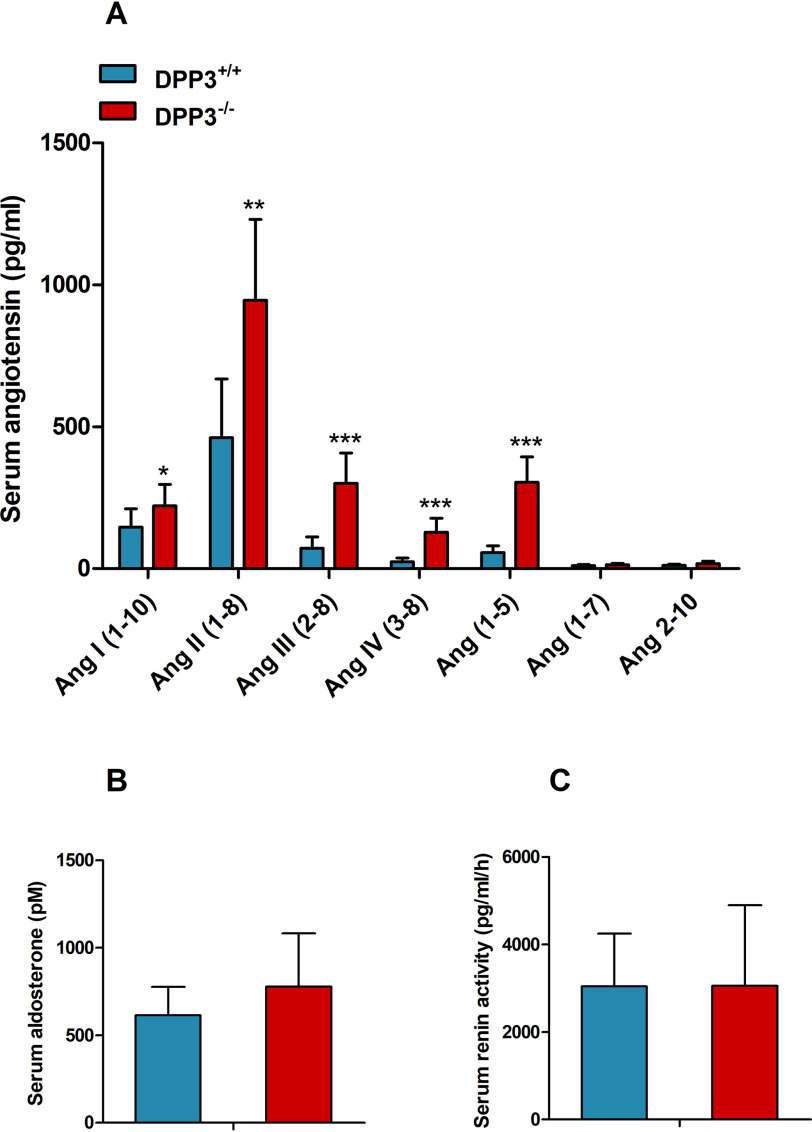
**DPP3^**−/−**^ mice exhibit increased circulating angiotensin metabolites.** *A–C*, concentration of RAS peptides (*A*), aldosterone (*B*), and renin (*C*) activity in serum measured by LC–MS in male DPP3^−/−^ and DPP3^+/+^ mice (12–16 weeks of age; *n* = 8/group). *, *p* < 0.05; **, *p* < 0.01; ***, *p* < 0.001 *versus* WT mice based on unpaired two-sided Student's *t* test. The data represent the means ± S.D.

Our observations indicate that DPP3 is involved in the regulation of water homeostasis, and recently published data suggested a role for DPP3 in angiotensin degradation (9, 21). Accordingly, we generated a serum “RAS fingerprint” consisting of 10 different angiotensin peptides using LC–MS/MS. Serum analysis revealed that male DPP3^−/−^ mice had higher concentrations of most angiotensin metabolites. As shown in Fig. 5*A*, the concentration of Ang II was twice as high as in WT mice. Interestingly, downstream peptides, in particular Ang(1–5), Ang III, and Ang IV, accumulated 5.4-, 4.2-, and 5.3-fold, respectively, suggesting that DPP3 has multiple natural substrates among angiotensin metabolites and that DPP3 deletion leads to perturbation of the entire RAS in males. Serum aldosterone levels (Fig. 5*B*) and serum renin activity (Fig. 5*C*) remained unchanged.

### Deletion of DPP3 enhances oxidative stress in male mice

**Figure 6.**
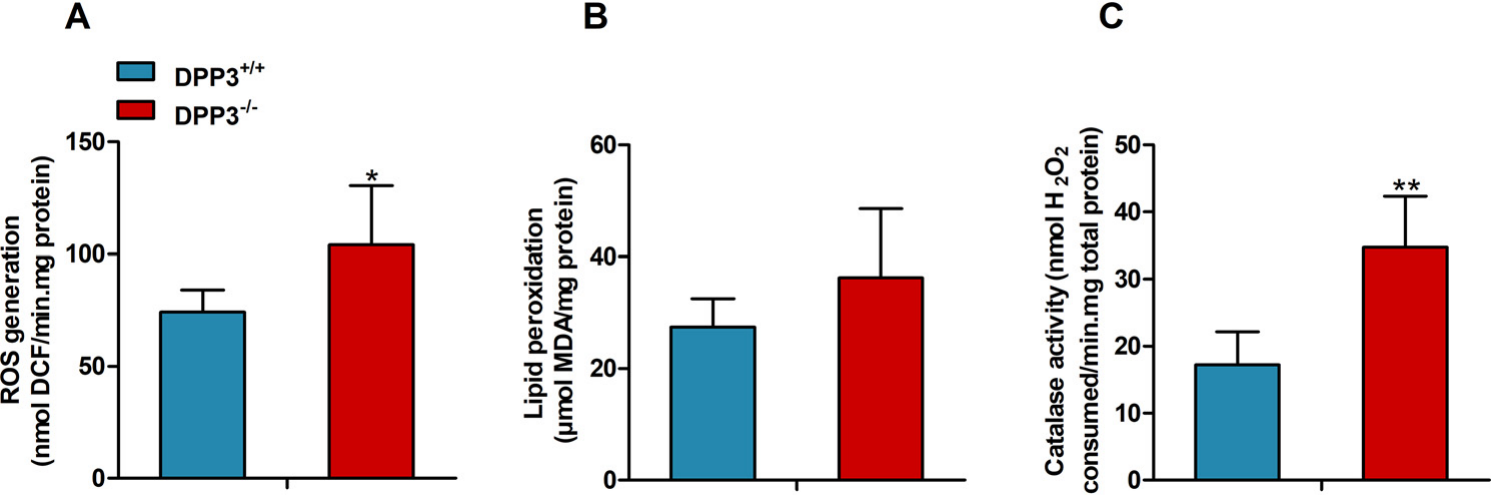
**DPP3 knockout renders mice susceptible to oxidative stress.** *A–C*, quantification of ***(***ROS production (*A*), lipid peroxidation (*B*), and catalase activity (*C*) in kidney homogenates of male DPP3^−/−^ and DPP3^+/+^ mice (12–16 weeks of age; *n* = 5/group). *, *p* < 0.05; **, *p* < 0.01 *versus* WT mice based on unpaired two-sided Student's *t* test. The experiments were performed in technical triplicates, and the data represent the means ± S.D.

**Figure 7.**
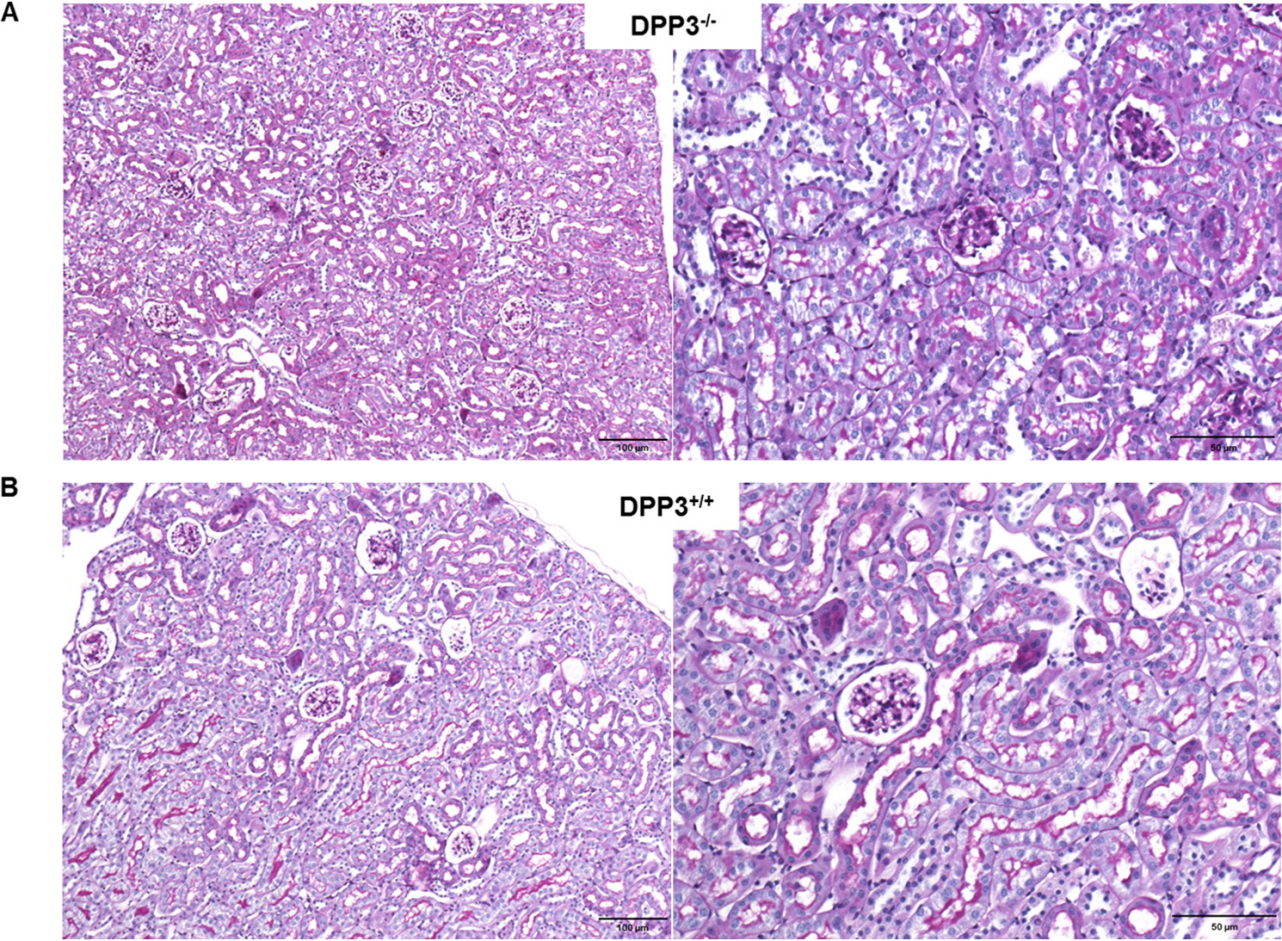
**DPP3 deletion does not affect morphology of mouse kidney.** Periodic acid–Schiff–stained slides of renal histology showing unremarkable glomeruli and tubuli in DPP3^−/−^ (*A*) and DPP3^+/+^ (*B*) mice. *Left panels*, 10×; *right panels*, 20×.

Ang II is known to promote ROS production in kidney, and therefore we determined the generation of reactive oxygen intermediates using 2′,7′-dichlorodihydrofluorescein diacetate (H_2_DCFDA). DPP3 deletion led to a significantly enhanced fluorescence signal from the ROS reporter dye H_2_DCFDA in the kidney lysates prepared from male mice (Fig. 6*A*). Accumulation of ROS in the DPP3^−/−^ kidneys also triggered a trend toward higher malondialdehyde levels, a marker of lipid peroxidation (Fig. 6*B*). Catalase activity was significantly increased in kidney homogenates of male DPP3^−/−^ mice, indicating increased generation of H_2_O_2_ (Fig. 6*C*). We also observed that despite this increase in renal ROS, the kidney morphology was not significantly altered between the genotypes (Fig. 7). We observed normal glomeruli with mesanglial normocellularity and thin basement of the capillary loops. Moreover, renal tubules and interstitium appeared normal, lacking signs of inflammation or fibrosis.

### DPP3 deficiency does not affect blood pressure

**Figure 8.**
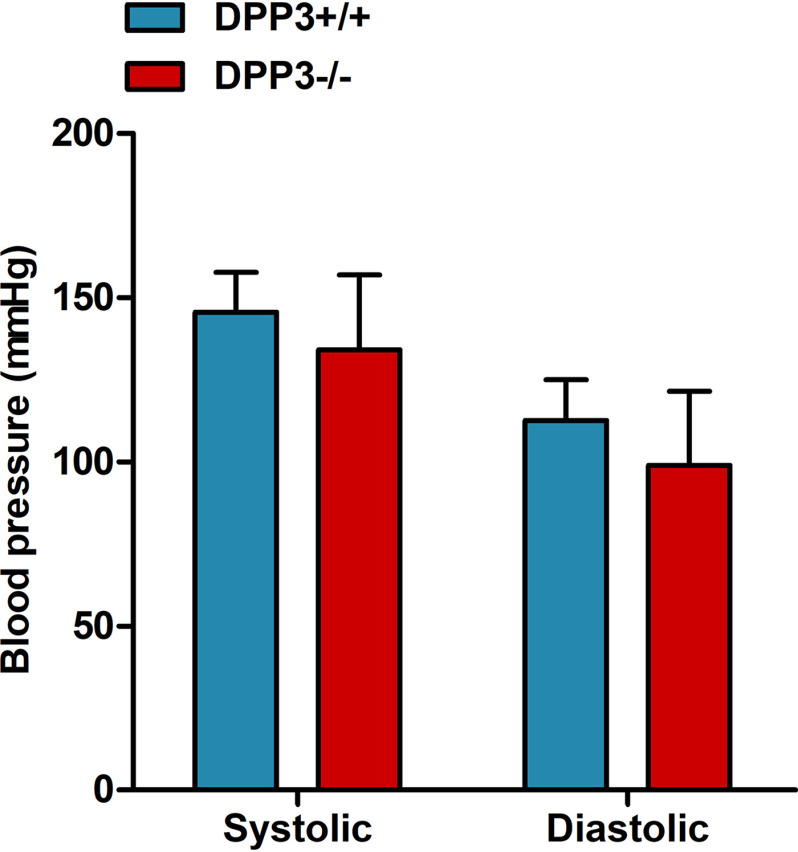
**DPP3 deficiency does not lead to changes in blood pressure.** Systolic and diastolic blood pressure were measured by tail-cuff method in male DPP3^+/+^ and DPP3^−/−^ mice (18–22 weeks of age; *n* = 8/group). The data are presented as the means ± S.D.

The extensive changes in the RAS accompanied by increased oxidative stress levels in kidneys of DPP3^−/−^ mice prompted us to assess potential effects on blood pressure, which is one of the major physiological output parameters of the RAS. Toward that end, we measured blood pressure in 18–22-week-old DPP3^+/+^ and DPP3^−/−^ mice by the tail-cuff method. There was no significant difference between DPP3^+/+^ and DPP3^−/−^ mice, both for the systolic (DPP3^+/+^, 145.6 ± 12.1 mm Hg *versus* DPP3^−/−^, 134.2 ± 22.7 mm Hg) and diastolic (DPP3^+/+^, 112.6 ± 12.4 mm Hg *versus* DPP3^−/−^, 99.0 ± 22.5 mm Hg) blood pressure (Fig. 8).

### Purified DPP3 catalyzes the turnover of multiple angiotensin peptides

**Figure 9.**
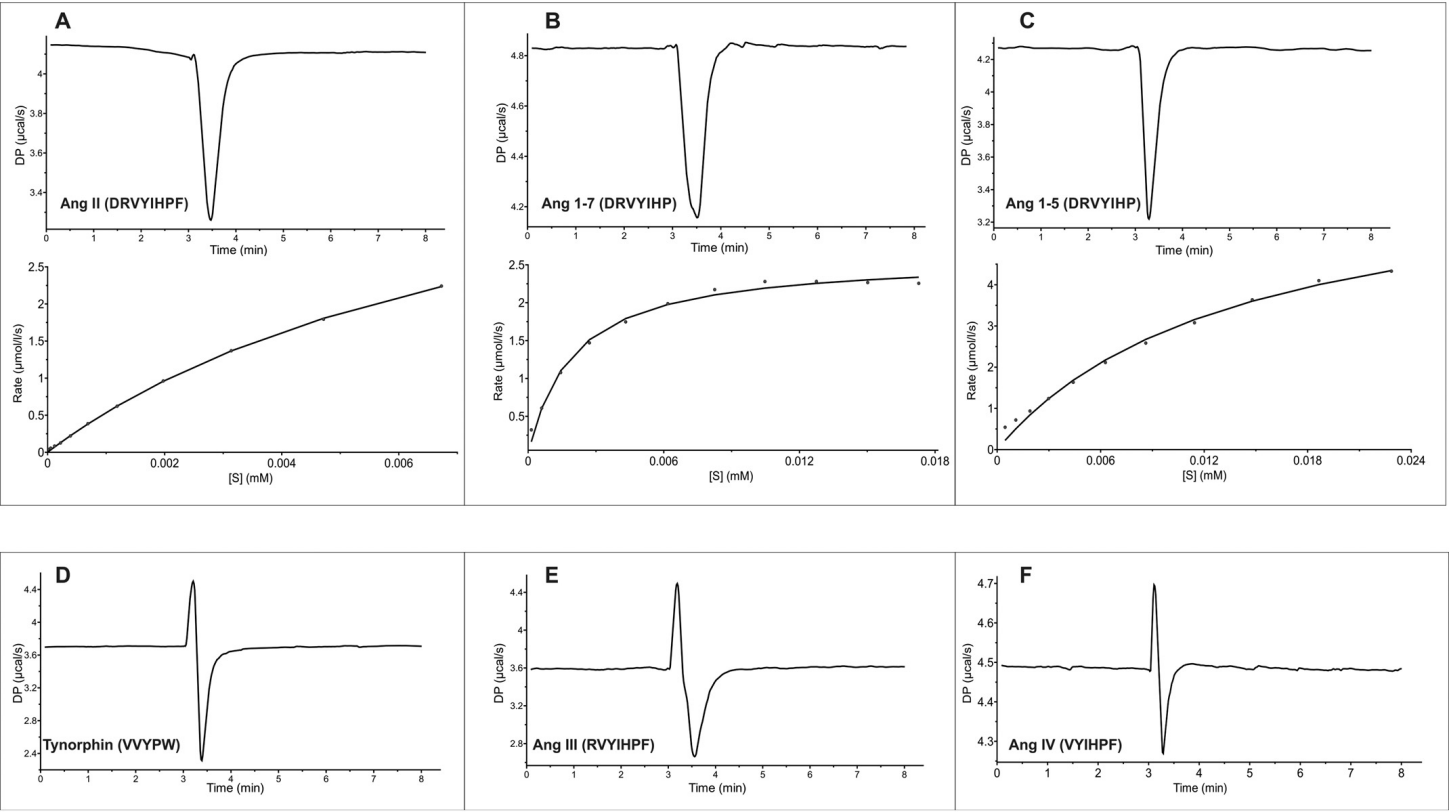
**RAS peptides can be “good” or “bad” substrates of DPP3.** RAS peptides turned over by DPP3 (*DP*), thus acting as “good” substrates (*A–C*). Raw data showing the heat change of the reaction as a function of time (*top panels*) and fitted curve for the rate of reaction (*bottom panels*) of Ang II (*A*), Ang(1–7) (*B*), and Ang(1–5) (*C*). Some RAS peptides demonstrated both endothermic and exothermic behavior, thus acting as “slow” substrates of DPP3 (*D–F*). The biphasic peaks were most likely due to binding to DPP3 and a subsequent slow turnover event. Raw data showing the heat change of the reaction in the case of slow substrates, tynorphin (*D*), Ang III (*E*), and Ang IV (*F*). Curve fitting was not possible in the case of slow substrates. The reaction was started by injecting 5 µl of 2 mm angiotensin peptides to the calorimetric cell containing 20 μm hDPP3. The data represent three or more technical replicates from two biological replicates.

The pleiotropic effect of DPP3 deletion on angiotensin peptides suggests that the enzyme not only accepts Ang II as a substrate but may also degrade other peptides in this pathway. Thus, we investigated the activity of DPP3 against different angiotensin peptides. Kinetic parameters of angiotensin peptides were obtained by single-injection calorimetry measurements that are based on the exothermal hydrolysis of peptide bonds by purified recombinant human DPP3, which shares 93% sequence identity with the mouse DPP3. Fig. 9 displays the curves for the rate of the reaction, calculated from integrated raw data using the enzyme kinetics–single-injection fitting model. The *graphs* show the rate of angiotensin conversion as a function of its concentration. The *insets* depict the raw data of heat change caused by the conversion of substrate peptides. The dependence of the reaction rate on angiotensin concentration followed typical Michaelis–Menten kinetics, and the parameters—enthalpy heat change (Δ*H*), turnover number (*k*_cat_), and *K_m_*—were found by the fitting model in the MicroCal PEAQ-ITC analysis software (Table 1). Among the six angiotensin peptides tested, Ang I, Ang II, Ang(1–5), and Ang(1–7) showed exothermic reactions, indicating their turnover by purified hDPP3 (Fig. 9, *A–C*). However, in the case of Ang I, data extraction was not possible because of the weak exothermic signal observed, indicating that Ang I is a poor substrate of DPP3. In contrast, Ang III and Ang IV displayed both endothermic and exothermic behavior, a characteristic observed for slow substrates like the peptide tynorphin and its derivatives (Fig. 9, *D–F*) (5). These experiments clearly confirm that DPP3 not only acts on Ang II but also efficiently hydrolyzes Ang(1–5) and Ang(1–7).

**Table 1:** **Kinetic parameters obtained from single-injection ITC experiments with hDPP3 and angiotensin peptides** The Michaelis–Menten constant (*K_m_*) and maximum turnover number (*k*_cat_) are obtained from the fit to a Michaelis–Menten curve for the reaction rate as a function of substrate concentration. The enthalpy of interaction (Δ*H*, also called heat of reaction) is a direct measurement of the rate at which heat is exchanged with the surroundings. The data represent the means ± S.D. All data points are three or more technical replicates from two biological replicates.

	Kinetic, calorimetric parameters of hDPP3 and angiotensin interaction		
	Δ*H*	*k*_cat_	*K_m_*
	*kcal/mol*	*s*^−*1*^	μ*m*
Ang II	−1.97 ± −0.06	0.25 ± 0.007	8.40 ± 0.40
Ang(1–7)	−1.52 ± −0.10	0.14 ± 0.003	1.95 ± 0.20
Ang(1–5)	−1.84 ± −0.04	0.35 ± 0.002	12.50 ± 2.20

### DPP3 deficiency has only minor impact on the phenotype of female mice

**Figure 10.**
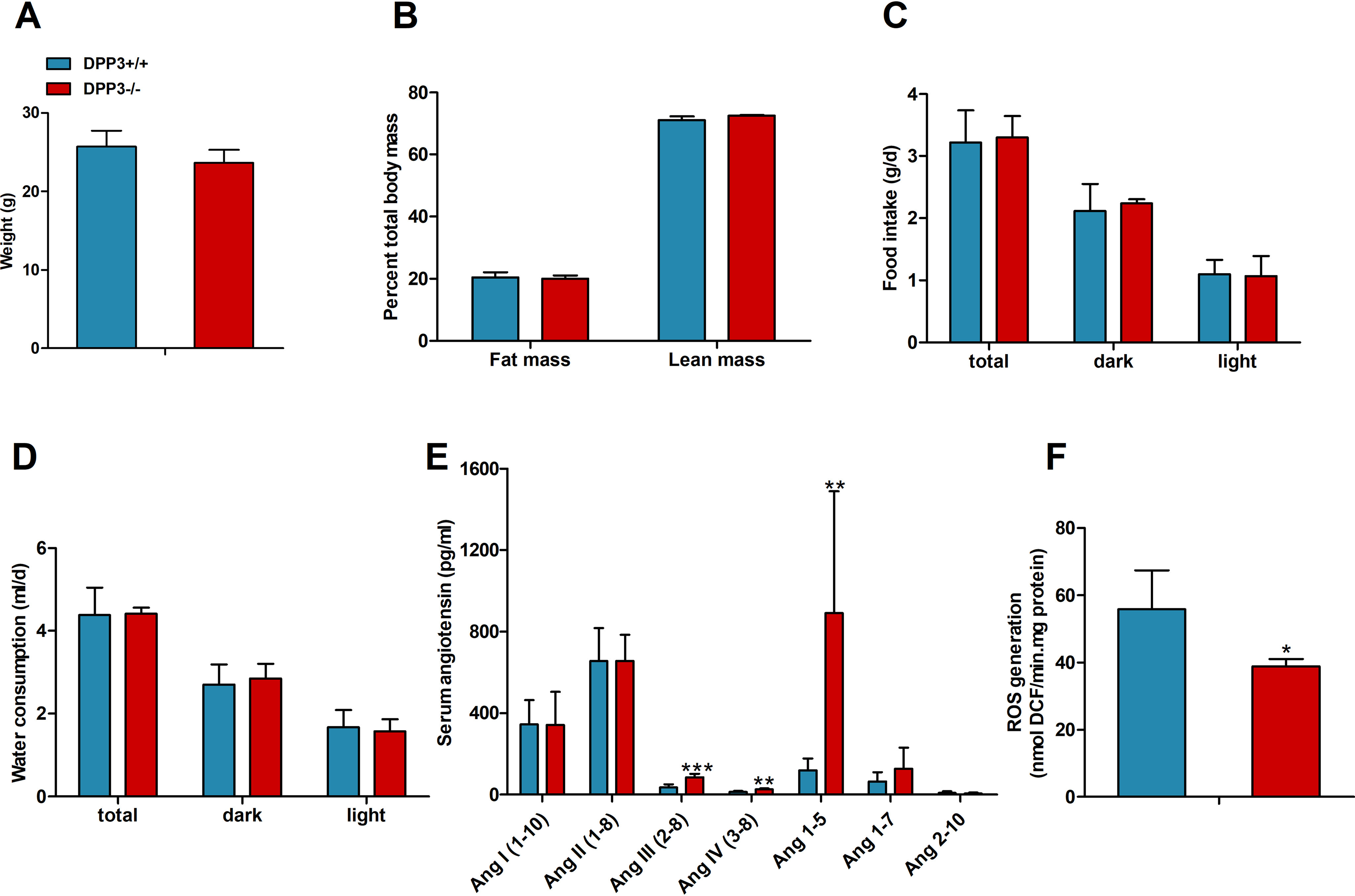
**DPP3 exerts a sex-specific effect on the knockout mice.** *A*, body weight (*A*) and body composition of female mice (*B*) fed a regular chow diet. *C* and *D*, daily food intake (*C*) and water consumption (*D*) was measured in metabolic cages over the light and dark phases in DPP3^−/−^ and DPP3^+/+^ female mice (12–16 weeks of age; *n* = 6/group) fed a regular chow diet over a period of 6 consecutive days. *E*, concentration of RAS peptides in serum measured by LC–MS in DPP3^−/−^ and DPP3^+/+^ female mice (12–16 weeks of age; *n* = 8/group). *F*, quantification of ROS production in DPP3^−/−^ and DPP3^+/+^ female mice (12–16 weeks of age; *n* = 5/group). *, *p* < 0.05; **, *p* < 0.01 *versus* WT mice based on unpaired two-sided Student's *t* test. The data represent means ± S.D.

In contrast to male mice, female DPP3^−/−^ mice did not show any significant difference in body weight (Fig. 10*A*) or body mass composition (Fig. 10*B*). Moreover, food intake (Fig. 10*C*) and water consumption (Fig. 10*D*) were not different between the genotypes. Most importantly, serum levels of Ang II remained unaltered in female DPP3^−/−^ mice as compared with WT, whereas the levels of Ang III, Ang IV, and Ang(1–5) were 2.4-, 2-, and 7.5-fold higher, respectively (Fig. 10*E*). Contrary to male DPP3^−/−^ mice, the amount of ROS generated was significantly lower in the kidney lysates of DPP3^−/−^ female mice, indicating sex-specific differences (Fig. 10*F*).

## Discussion

In this study, we demonstrate that deletion of *DPP3* in mice caused widespread physiological and biochemical changes. In addition to the previously observed alterations in body weight and bone morphology (11), the lack of DPP3 clearly affects the RAS pathway, which is associated with increased water consumption. This is characteristic for mouse models lacking genes associated with the RAS like ACE or angiotensinogen, because they have diminished ability to concentrate urine due to impaired renal development (22, 23). However, we found no obvious changes in kidney morphology, indicating that, at least in the young mice investigated in this study, renal function is normal.

Male DPP3^−/−^ mice displayed increased levels of equilibrium angiotensin peptides including Ang II as well as a specific qualitative shift in angiotensin metabolite profiles, characterized by selective and profound increases of Ang(1–5), Ang III, and Ang IV. These profound differences in angiotensin profiles indicate that a general up-regulation of the RAS at the level of renin can be excluded as an underlying cause for the observed Ang II increase. Ang II is an integral part of the RAS and mediates various physiological responses. High Ang II levels increases reactive oxygen species and oxidative stress and depresses mitochondrial energy metabolism (15, 16, 18, 19, 24). There are several reports suggesting that high circulating Ang II concentration is a stimulus for thirst in a variety of species, which is consistent with our findings in male DPP3^−/−^ mice (14, 25, 26). Because Ang II (16, 18, 24, 27, 28) mediates ROS production, which can contribute to oxidative stress, we measured putative stress markers in the kidneys of mice. Congruously, the level of ROS generation and catalase activity were elevated in the knockouts. These results confirm our hypothesis that increased Ang II creates oxidative stress in male DPP3^−/−^ mice.

In addition to regulating stress response via modulation of Ang II, it is likely that DPP3 is part of endogenous defense system against oxidative stress. DPP3 is known to promote nuclear migration of transcription factor Nrf2 by displacing Keap1 (10, 29). The Keap1–Nrf2 pathway is a key regulator of cellular stress response caused by ROS. Under basal conditions, Nrf2 is bound to Keap1. Upon activation by oxidative stressors, Nrf2 translocates to the nucleus of the cell where it activates the ARE, thereby regulating the transcription of genes responsible for antioxidant and anti-inflammatory defense (30–33). It has been reported that overexpression of DPP3 could potently activate the ARE in neuroblastoma cells (IMR-32 cells), leading to increased expression levels of NAD(P)H:quinone oxidoreductase 1 (NQO1), a phase II detoxifying enzyme regulated by ARE. It was also found that DPP3 overexpression efficiently attenuated the toxic effects of H_2_O_2_ and rotenone, demonstrating the cytoprotective effect of DPP3 against oxidative stress (34). Furthermore, it was demonstrated that the DPP3 plasma levels are associated with the survival rate of patients suffering from sepsis (35), cardiogenic shock (36), heart failure (37), and acute kidney injury (38). Our results are also consistent with a recent study reporting that a lack of DPP3 leads to impaired bone homeostasis and enhanced oxidative stress in the bone tissue (11), indicating a ubiquitous antioxidant activity of DPP3.

Increase in the Ang II levels leads to vasoconstriction and, thereby, severe hypertension (17, 19, 27). However, both male and female DPP3^−/−^ mice showed no change in the blood pressure using the tail-cuff method under normal dietary conditions. Because measuring blood pressure using the tail-cuff method has certain limitations (*e.g.* stress response of mice), further studies will be necessary to investigate a potential fine tuning of blood pressure regulation in DPP3^−/−^ mice. The unchanged blood pressure also points toward the involvement of the cardioprotective arm of RAS as a compensatory mechanism (8). *In vitro* studies using recombinant human DPP3 additionally identified Ang(1–7) and Ang(1–5) as substrates of the enzyme. Although the level of Ang(1–7) was not significantly increased in the knockout, it is interesting to note that it is the main metabolite of the alternate RAS and plays an important role as physiological antagonist of Ang II, having vasodilatory and antihypertensive properties (8). Ang(1–7) is produced by carboxyl- or endopeptidases like neutral endopeptidase, prolyl endopeptidase, ACE2, or prolyl carboxypeptidase from Ang I and Ang II and is metabolized to Ang(2–7) and Ang(3–7) by aminopeptidases and to Ang(1–5) by ACE. In contrast to Ang(1–7), Ang(1–5) showed a very pronounced change between WT and knockout mice exhibiting a more than 5-fold higher concentration in the latter. The specific increase of Ang(1–5) in DPP3^−/−^ mice points to a clear preference of DPP3 toward Ang(1–5) over Ang(1–7). It was shown that Ang(1–5) stimulated the secretion of atrial natriuretic peptide via Mas receptor, a mode of action similar to that of Ang(1–7) (39). The atrial natriuretic peptide system is a hormonal system that participates in the regulation of body fluid and electrolyte balance. It acts antagonistically to the RAS and causes anti-hypertension and anti-oxidative stress (40, 41). It is conceivable that Ang(1–5) also plays a role as a vasodilatory and antihypertensive peptide.

Female DPP3^−/−^ mice displayed a less pronounced phenotype compared with male mice, indicating sex-specific differences. Accumulating evidence suggests that there are sex differences in the tissue expression and activity of several RAS components, with the female sex hormone, estrogen, down-regulating Ang II and upregulating Ang(1–7) pathways (42). It was shown that in C57BL/6J mice, the Ang II-induced increase in blood pressure is greater in males than in females (42). Also, healthy men had greater pressor and renal vasoconstrictor responses to acute Ang II infusion compared with women (43). Similarly, chronic Ang II infusion induced hypertension in male but not female mice (44, 45), possibly because of a shift in the balance from Ang II toward Ang(1–7) pathways because of estrogen-mediated protection (46). In high fat–fed C57BL/6J mice, females maintained circulating Ang(1–7) levels and were protected from hypertension and metabolic complications induced by Ang II (47). These studies are in line with our observations from the serum RAS fingerprint, which show that the female DPP3^+/+^ and DPP3^−/−^ mice had 6- and 9-fold higher Ang(1–7), respectively, than the male mice. For Ang(1–5), the levels were 2- and 3-fold higher than in male DPP3^+/+^ and DPP3^−/−^ mice, respectively. This is indicative of an up-regulation of the protective RAS arm in female mice.

On a similar note, numerous studies have discussed the association of DPP3 to estrogen. It was reported that 17β-estradiol (E2), the predominant estrogen present in the serum, influences the expression level of DPP3 *in vivo*. The hepatic DPP3 levels were found to be markedly reduced following ovariectomy in 16-week-old CBA/H mice and E2 administration abolished this effect and increased DPP3 protein expression (48). It was also found that DPP3 is instrumental in estrogen-mediated protection against oxidative stress in female CBA/H mice (49). DPP3 accumulated in the nucleus in liver tissue lysates of healthy female mice exposed to hyperoxia, at levels comparable with the nuclear accumulation of Nrf2. Further, the combined induction of hyperoxia and E2 administration had a synergistic effect on the nuclear accumulation of DPP3. In ovariectomized females exposed to hyperoxia, supplementation of E2 enhanced DPP3 levels, accompanied by an up-regulation of other cytoprotective proteins like sirtuin-1 and heme oxygenase-1, resulting in attenuated oxidative stress (49). Lack of DPP3 was found to augment bone loss caused by estrogen deprivation in the ovariectomized mouse model of human postmenopausal osteoporosis (11). This interplay between DPP3, RAS, and estrogen demands additional studies; however, it is very likely that the female DPP3^−/−^ mice are protected from oxidative stress by the presence of estrogen. The male DPP3^−/−^ mice on the other hand show an aggravated response to Ang II–induced oxidative stress because of this lack of antioxidant and cytoprotective function.

In summary, the generation and characterization of a mouse model with global deletion of DPP3 has revealed a significant perturbation of the levels of peptides in the RAS. The changes in peptide levels were found to be associated with polydipsia and augmented levels of ROS. Our findings identify DPP3 as a pleiotropic and sex-specific modulator of RAS and emphasize its role in oxidative stress response.

## Experimental procedures

### Ethics statement

All animal experiments were approved by the Austrian Federal Ministry for Science, Research, and Economy (protocol number BMWF-66.007/7-ll/3b/), the ethics committee of the University of Graz, and conducted in compliance with the Council of Europe Convention (ETS 123).

### Animals and generation of DPP3 knockout mice

All studies were conducted in age-matched DPP3^−/−^ and WT control male and female mice on C56BL/6J background. Unless stated otherwise, the results describe the effects of DPP3 deletion on male mice. The mice were bred and maintained at regular housing temperatures (23 ± 1 °C) with a 14-h light/10-h dark cycle. The animals had *ad libitum* access to water and chow diet (4.5% fat, 34% starch, 5.0% sugar, and 22.0% protein; Ssniff Spezialdiaeten). Breeding and genotyping were done according to standard procedures. For generation of DPP3^−/−^ mice, targeted mutant ES cells were obtained from EUCOMM and injected into blastocysts of C57BL/6J mice. Chimeric animals with a high degree of coat color chimerism were bred with C57BL/6J mice. The construct containing a β-galactosidase cassette (lacZ) and a promotor-driven selection cassette (neo) was inserted into the *DPP3* gene. Additionally, the construct contained two flippase recognition target sites for flippase recombination enzyme–mediated recombination flanking lacZ and neo. The selection cassette and exon 6 (essential for DPP3 function), flanked by loxP sites, were removed by breeding with transgenic C57BL/6J mice expressing cre-recombinase under the control of a CMV promotor (CMV-Cre). Cre-lox recombination resulted in deletion of neo and exon 6, leaving the lacZ reporter gene intact. Mice totally lacking *DPP3* were bred by crossing mice heterozygous for the mutant *DPP3* allele lacking neo and exon 6.

### Serum and tissue lysate preparation

The animals were anesthetized with isoflurane, and blood was collected by the retro-orbital puncture. Immediately following collection, the blood was allowed to clot, and serum was isolated by centrifugation. For tissue collection, mice were sacrificed by cervical dislocation and tissues were surgically removed and washed with cold PBS. Homogenization was performed on ice in solution A (0.25 m sucrose, 1 mm EDTA, 20 μm DTT, 0.1% Triton X-100, 20 μg/ml leupeptin, 2 μg/ml antipain, 1 μg/ml pepstatin, pH 7.0) using an Ultra Turrax (IKA, Staufen, Germany). 20,000 g infranatant was used for further experiments. Protein concentrations in the tissue lysates were estimated using a protein assay dye reagent from Bio-Rad using BSA as standard. Serum and tissue samples were stored at −80 °C until further analysis.

### SDS-PAGE and Western blotting

Tissue lysates were diluted in Laemmli's sample buffer, and 20 µg of total protein/lane was subjected to SDS-PAGE using 10% SDS–polyacrylamide gels. The resolved proteins were transferred onto polyvinylidene difluoride membranes (VWR, Radnor, PA, USA) using a Trans-Blot SD transfer cell (Bio-Rad). Following transfer, the membranes were washed with TBS containing 0.01% Tween 20 (TBST) and then blocked in 5% nonfat milk for 1 h at room temperature. The membranes were then incubated overnight with anti-DPP3 rabbit polyclonal antibody (1:1,500; Proteintech Europe, Manchester, UK) in TBST containing 5% nonfat milk at 4 °C. After washing three times for 10 min in TBST, the membranes were incubated with peroxidase-labeled secondary antibody (1:5,000; Cell Signaling Technology, Danvers, MA, USA) for 1 h at room temperature. The immunoblots were developed using enhanced chemiluminescent Western blotting substrate solution (Pierce–Thermo Fisher Scientific).

### DPP3 activity assay in mouse tissue lysates

DPP3 activities in tissue lysates were determined by fluorometrically (excitation, 332 nm; emission, 420 nm) measuring the liberation of 2-naphthylamine at 37 °C in a mixture containing 25 µl of 200 μm Arg-Arg-2-naphthylamide as substrate in TBS buffer (50 mm Tris, 100 mm NaCl, pH 8.2) and tissue lysate equivalent to 20 µg of total protein in a reaction mixture of 235 µl (white, tissue culture treated Krystal 2000 96-well plate from Porvair Sciences, Norfolk, UK). The activity assay was performed by continuous measurement of fluorescence of 2-naphthylamine for 30 min (fluorescent plate reader from Molecular Devices, Sunnyvale, CA, USA). The reaction was started by the addition of the substrate. The samples were measured in triplicate.

### Body composition and metabolic phenotyping

The lean and fat masses of mice were analyzed by NMR (Minispec; NMR Analyzer, Bruker, Ettlingen, Germany). To measure spontaneous physical activity, O_2_ consumption, CO_2_ production, and food and water intake, the mice were housed in metabolic cages allowing continuous measurement of these parameters (LabMaster; TSE Systems GmbH, Bad Homburg, Germany). For measurements of energy balance, the animals were familiarized with these cages for at least 72 h before data collection.

### Analysis of angiotensin peptides in serum

Serum equilibration was performed at 37 °C followed by stabilization of equilibrium angiotensin levels and subsequent quantification by LC–MS/MS analysis (50). Briefly, stable isotope-labeled internal standards for each Ang metabolite (Ang I (1–10), Ang II (1–8), Ang(1–7), Ang(1–5), Ang III (2–8), Ang IV (3–8), Ang(1–9), Ang(3–7), Ang(2–7), and Ang(2–10)) were added to stabilized serum samples at a concentration of 200 pg/ml. Following C18-based solid-phase extraction, the samples were subjected to LC–MS/MS analysis using a reversed-phase analytical column (Acquity UPLC^®^ C18, Waters) operating in line with a XEVO TQ-S triple quadrupole mass spectrometer (Waters Xevo TQ/S, Milford, MA, USA) in multiple reaction monitoring modes. The internal standard was used to correct for analyte recovery across the sample preparation procedure in each sample. Analyte concentrations were calculated from integrated chromatograms considering the corresponding response factors determined in appropriate calibration curves in serum matrix.

### Determination of ROS generation

The intracellular ROS level was detected by using H_2_DCFDA (Sigma–Aldrich). When oxidized by various active oxygen species, it is irreversibly converted to the fluorescent form, 2′,7′–dichlorofluorescein (DCF) (51, 52). ROS in kidney tissue was estimated by diluting tissue lysate equivalent to 100 µg of total protein in ice-cold 40 mm Tris-HCl buffer (pH 7.4). The samples were divided into two equal fractions. In one fraction, 40 µl of 10 μm H_2_DCFDA in methanol was added for ROS estimation. Another fraction with 40 µl of methanol was used as a control for tissue auto-fluorescence. All of the samples were incubated at 37 °C for 15 min, and fluorescence was determined at 485-nm excitation and 525-nm emission using a fluorescence plate reader (Molecular Devices). To quantitate ROS levels, relative dichlorofluorescein (DCF) fluorescence was used as a standard.

### Detection of lipid peroxidation activity

The extent of lipid peroxidation in kidney was assessed using thiobarbituric acid (TBA) reactive substances as an index. For the assay, kidney tissue equivalent to 1 mg of total protein was incubated with 20% tricarboxylic acid and 0.67% thiobarbituric acid (TBA). The reaction mixture was heated at 100 °C for 30 min and then cooled in an ice-bath for 10 min. The samples were then centrifuged at 3,000 rpm for 15 min. The supernatant was collected to measure absorbance at 532 nm. The formation of thiobarbituric acid reactive substances was expressed using malondialdehyde equivalent as a standard.

### Catalase activity

Catalase activity is measured as described in Ref. 53. Briefly, kidney tissue equivalent to 1 mg of total protein in 0.01 m PBS was incubated with 0.2 m H_2_O_2_. The reaction was stopped by adding 5% dichromate solution at 30-s intervals. The samples were heated at 60 °C for 10 min where the blue precipitate formed was decomposed to a green solution. Consumption of H_2_O_2_ was determined by recording absorbance at 570 nm. A standard curve containing 0–100 μm of H_2_O_2_ was prepared to determine the amount of H_2_O_2_ present in each sample.

### Histological analysis of kidney

For the analysis of morphological differences, the kidneys were fixed in 4% neutral buffered formaldehyde solution for 24 h, embedded in paraffin, and further processed for periodic acid–Schiff staining (2-μm-thick sections).

### Blood pressure measurements

The CODA 8-channel noninvasive tail-cuff technique (Kent Scientific, EMKA Technologies, Paris, France) was used for blood pressure measurements in young adult male mice from 12 to 16 weeks of age. This system uses volume pressure recording to detect blood pressure based on volume changes in the tail (54). Volume pressure recording cuffs were checked routinely before the start of the experiments. The heating pads were preheated to 35 °C before and during the measurements. Each measurement consisted of five acclimation cycles followed by 15 cycles during which the systolic and diastolic pressure were measured. Acclimation cycles were not used in blood pressure analysis.

### Isothermal titration calorimetry

Calorimetric activity of six different angiotensin peptides as purported substrates of DPP3 were further assayed using a MicroCal PEAQ-ITC (Malvern Panalytical Ltd., Malvern, UK). The peptides used for this assay, Ang I (1–10), Ang II (1–8), Ang III (2–8), Ang IV (3–8), Ang(1–5), and Ang(1–7), were commercially purchased (Bachem, Bubendorf, Switzerland). For this, the single-injection method was used, in which 5 µl of 2 mm angiotensin peptide was titrated into 200 µl of 20 μm purified hDPP3. The purification of recombinant hDPP3 was done as described in Ref. 35. Both the ligand and the purified protein were prepared in 50 mm Tris-HCl at pH 8.0 containing 100 mm NaCl. The experiments consisted of a single injection of the ligand continuously over a period of 10 s, including an initial delay of 180 s before the injection and a spacing of 300 s after the injection. The experiments were performed at 25 °C with a stirring speed of 500 rpm. The thermodynamic characterization of this binding interaction was obtained using MicroCal PEAQ-ITC analysis software.

### Statistical analysis

All data are expressed as means ± S.D. The results were assessed using two-tailed unpaired Student's *t test* (GraphPad Prism 5, San Diego, CA, USA). A *P* value less than 0.05 was considered significant.

## Data availability

All data presented and discussed are contained within the article.
